# Movement-level process modeling of microsurgical bimanual and unimanual tasks

**DOI:** 10.1007/s11548-021-02537-4

**Published:** 2021-12-15

**Authors:** Jani Koskinen, Antti Huotarinen, Antti-Pekka Elomaa, Bin Zheng, Roman Bednarik

**Affiliations:** 1 School of Computing, University of Eastern Finland, 80110 Joensuu, Finland; 2 Department of Neurosurgery, Institute of Clinical Medicine, Kuopio University Hospital, 70211 Kuopio, Finland; 3 Microsurgery Center, Kuopio University Hospital, 70211 Kuopio, Finland; 4 Surgical Simulation Research Lab, Department of Surgery, University of Alberta, Edmonton, AB Canada

**Keywords:** Microsurgery, Surgical process modeling, Bi-manual dexterity, Surgical education

## Abstract

**Purpose:**

Microsurgical techniques require highly skilled manual handling of specialized surgical instruments. Surgical process models are central for objective evaluation of these skills, enabling data-driven solutions that can improve intraoperative efficiency.

**Method:**

We built a surgical process model, defined at movement level in terms of elementary surgical actions ($n=4$) and targets ($n=4$). The model also included nonproductive movements, which enabled us to evaluate suturing efficiency and bi-manual dexterity. The elementary activities were used to investigate differences between novice ($n=5$) and expert surgeons ($n=5$) by comparing the cosine similarity of vector representations of a microsurgical suturing training task and its different segments.

**Results:**

Based on our model, the experts were significantly more efficient than the novices at using their tools individually and simultaneously. At suture level, the experts were significantly more efficient at using their left hand tool, but the differences were not significant for the right hand tool. At the level of individual suture segments, the experts had on average 21.0 % higher suturing efficiency and 48.2 % higher bi-manual efficiency, and the results varied between segments. Similarity of the manual actions showed that expert and novice surgeons could be distinguished by their movement patterns.

**Conclusions:**

The surgical process model allowed us to identify differences between novices’ and experts’ movements and to evaluate their uni- and bi-manual tool use efficiency. Analyzing surgical tasks in this manner could be used to evaluate surgical skill and help surgical trainees detect problems in their performance computationally.

## Introduction

Small-scale procedures performed using a surgical microscope require a high degree of uni- and bi-manual dexterity, which thus form an essential part of surgical expertise. Surgical expertise has traditionally been assessed using mentor-trainee methods that suffer from subjectivity and time consumption issues [3, 25, 28] of human observers. With advances in computational power and the emergence of new sensing technologies, the focus has turned to developing objective computer-assisted evaluation methods. [25]

**Fig. 1:**
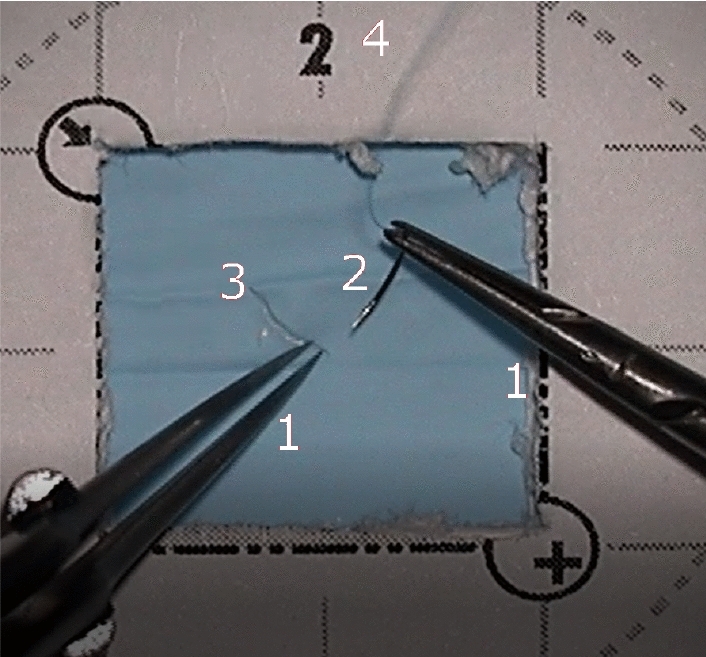
Frame from one of the recorded videos, showing the four targets: tools (1) (left: microforceps, right: needleholder), the needle (2), the incision (3) and the thread (4). In this example, the activity label for the needleholder would be <transport;incision> since it is transporting the needle to the incision, while the microforceps would be labeled with <hold still; incision>

**Table 1:** Suture segments with explanations. The surgical task also included cutting the ends of the thread after the three knots had been completed, but this part was left out of the analysis

Order	Suture segment	Description
1	Needle transport	A needle is picked up and transported to the incision
2	Piercing	The needle pierces the incision on both sides
3-5	Knot 1-3	Three surgical knots with differing number of loops are completed

A central requirement toward this goal has been the development of surgical process modeling. [17] Surgical process models are defined, for example, in terms of the actions of the surgeon or the surgical team. [21–23] An early work of MacKenzie et al. [19] showed a way to decompose a surgical procedure into a sequence of higher- and lower-level activities. The level of abstraction at which the activities are defined is called *granularity*. [11, 23] The granularity of the model can be defined in different ways. For example, MacKenzie et al. defined the surgical procedure in terms of steps, sub-steps, tasks and sub-tasks. Likewise, several different approaches have been used to define the model structure. In addition to the hierarchical scheme used by MacKenzie et al., another approach that has been used by several authors is to define the surgical activities as ordered lists (or n-tuples) of surgical actions, anatomical structures and instruments. (see, for example [5, 8, 26]).

Surgical process modeling enables the quantitative analysis of surgical processes. Such analysis, in turn, enables more objective classification of surgical expertise [17], comparison of surgical procedures [6], evaluation of learning curves [9], context-dependent support [21], prediction of surgeon’s actions [7] and the remaining intervention time. [10] Altogether, surgical process models are a precursor for intelligent surgical systems.

Here, we expand on the previous approaches to surgical process models that modeled the activities as n-tuples. [5, 8, 26] First, we define the surgical activities using only a few elementary actions and targets, with the aim of discovering if a surgical process model defined in such terms can still reveal differences in the participants’ microsurgical skills. As one of the activities, we include nonproductive movements, which allows us to evaluate participants’ efficiency. We then combine the annotation of elementary surgical activities with a segmentation of the surgical task into phases. By applying the surgical process model to video recordings of microsurgical training tasks, we can compare the similarity and efficiency between performances during the whole task and within the related segments, and thus evaluate overall differences between participants, and to discover the segments where the participant’s performance deviated the most.

We investigate if the surgical process model can be applied to extract sufficient information from a microsurgical training task to evaluate if: (1) expert surgeons will use their tools more efficiently than novices, (2) expert surgeons will display a higher level of similarity among surgical segments than novices and (3) experts’ will more frequently use their tools bi-manually than novices do in the microsurgical tasks.

## Methods and materials

### Experiment

Eleven participants were grouped into novices and experts. The experts ($n=6$) were plastic surgeons who were performing 30–60 monthly surgical operations using microscopes or loupes, whereas novice participants ($n=5$) had medical training but no clinical experience in microsurgical techniques. The experiment was approved by a local ethics committee and conducted in accordance with the Declaration of Helsinki.

The experiment was conducted in a surgical simulation laboratory. The participants completed 12 sutures on a microsurgical training board. The board had two rows of 3 boxes, each lined with a latex skin that had a pre-cut incision for making the suture (Fig. 1). Before starting the experiment, the participants were given instructions and asked to sign a consent form.

The participants completed the sutures using microsurgical needle holders and suturing forceps with 9.3 mm 3/8 taper head needles attached to 7–0, 50 cm prolypropylene monofilament sutures. One expert and one novice participant were left handed, but all participants held the microforceps in their left hand and the needleholder in their right hand. Microscope used in the experiment was a Zeiss OPMI Vario S88 and it was equipped with a camera for recording the scene under the microscope.

**Fig. 2:**
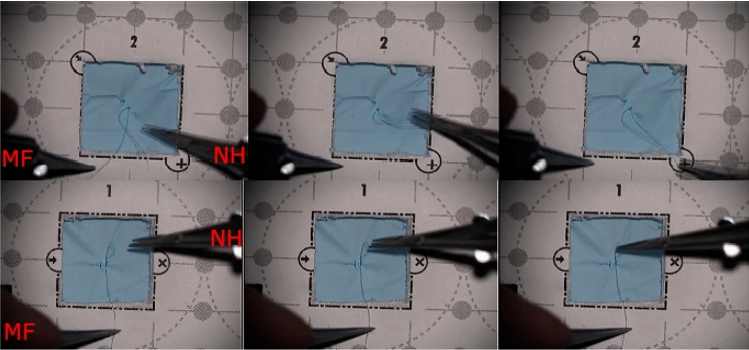
Examples of nonproductive movements with the microforceps (MF). *First row* Microforceps are waiting while the needleholder (NH) attempts to grasp the thread. *Second row* Microforceps are holding the end of the thread and waiting while the needleholder is attempting to grasp the other end

### Surgical process model

**Fig. 3:**
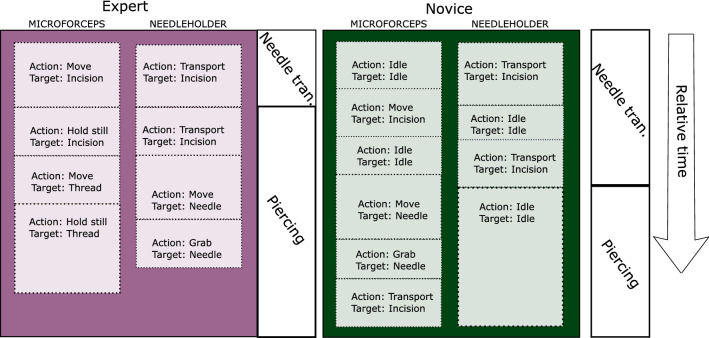
Example of the surgical process model description of the sutures for an expert and a novice. The figure shows the first two segments in terms of their <action;target> pairs. The expert transports the needle to the incision with the right hand while moving the left hand to the incision to support it while the needle is being pierced. The novice on the contrary fails to use both hands efficiently and has many movements without target

Our surgical process model was developed as a combination of top-down and bottom-up approaches. First, an expert neurosurgeon split the sutures into segments (Table 1). Then, we defined a set of activities in terms of elementary surgical actions and targets, and used them to describe the segments (Fig. 3). We annotated the videos manually, labeling each frame with action–target pairs for both hands. The surgeons’ left- and right-hand tool movements were labeled separately.

The activities were defined similarly to Forestier et al. [5, 7], who defined the surgical activities as n-tuples, a mathematical term to describe a construction of a series of ordered elements, including anatomical structures, surgical actions and instruments. We replaced the anatomical structure with a general class of targets consisting of the tool, the needle, the thread and the incision.

For the actions, we created four possible categories: move, transport, grab and hold still. Transport and movement are distinct because in the former, the operator must control the force used to handle the tool. The different labels are displayed in Table 2.

When the tool was not doing anything meaningful to advance the suture, the activity was labeled having *no target* and the movements were considered nonproductive. This commonly occurred when one tool was waiting for the second tool to complete some activity, see Fig. 2 for examples.

The activities were annotated by one of the authors. First, we determined a verbal description of what the tool is doing. The description could, for example, be, ”transporting the needle to the the incision,” or ”grasping the thread.” From these descriptive sentences, we would identify the action (”transport” in the first example and ”grasp” in the second) and the target of the action (”incision” in the first example and ”thread” in the second). Very short activities were merged with the previous activity, such as if the tool paused briefly (<0.5 s) during transportation. Likewise, short actions that in previous studies have been described with separate verbs were merged with the previous activity. For example, at the start of the suture the participant transports the needle to the incision and pierces the latex surface on both sides of the incision. This entire movement, until the needle is released, was described with the activity $<transport;incision>$.

In the <action;target> tuples, target is understood to be a ”target of interest,” i.e. the action does not necessarily imply movement toward the target. For example, when the thread is being extracted after piercing, the goal is to transport the thread away from the incision, so the surgical action is transport, the target is incision and the label is <transport;incision>. Altogether, there are 18 possible activities (4 actions x 4 targets + nonproductive movements + tool not visible), although some activities such as <grab;tool> never occurred (Table 2).

**Table 2:** Terminology used in the surgical process model for defining the surgical activities $\widehat{ac}_{kl}$. Because the tools remained the same, the surgical activities are defined by the actions and targets

Tools	Actions	Targets
Microforceps	Move	Needle
Needleholder	Transport	Incision
Not visible	Hold still	Thread
	Grasp	Tool
		No target/idle time

The surgical activities are considered as basis vectors, which are used to define vectors

1 $$\begin{aligned} \mathbf {sv_k}&= \begin{bmatrix} \beta _1 \widehat{ac}_{k1},&\ldots ,&\beta _n \widehat{ac}_{kn} \end{bmatrix} \end{aligned}$$

The component $\beta _l$ is the duration of the elementary surgical activity $\widehat{ac}_{kl}$, normalized so that the length of the vector $\mathbf {sv_k}$ is 1. At segment level, we obtained a vector for each segment in Table 1, and at suture level, we obtained one vector for each suture.

### Similarity measure and classification

The similarity between two segment vectors is defined to be the *cosine similarity*,

2 $$\begin{aligned} \cos (\theta ) = \frac{\mathbf {sv_i} \cdot \mathbf {sv_j}}{||\mathbf {sv_i}|| ||\mathbf {sv_j}||}, \end{aligned}$$

or the projection of the vector $sv_i$ on $sv_j$. Since all vectors have length 1 by definition, the similarity between two segments or sutures is the dot product of their respective vectors. The similarity was calculated separately for left and right hand tools.

For each suture, we calculated the similarity to every other suture in the dataset, excluding the participant’s own sutures. Then, we calculated each suture’s mean similarity to novice and expert sutures. In other words, each suture has two similarity values: a mean similarity to novices, and a mean similarity to experts. Whether the suture was closer to experts or novices was determined by subtracting the novice similarity from expert similarity: more negative values indicate closer similarity to novices, whereas positive values indicate closer similarity to experts. We compare the similarity at suture and segment levels independently for the microforceps and the needleholder.

### Efficiency measures

Having included the nonproductive movements as possible surgical activities, we can define suturing efficiency as the ratio of time spent on useful movements and total time:

3 $$\begin{aligned} S_{eff}=\frac{T- t_w}{T} \end{aligned}$$

where *T* is the total task duration and $t_w$ the time spent on nonproductive movements, i.e. the surgical activities that had no clear targets or when the tool was not visible.

Similarly, we can define bimanual efficiency

4 $$\begin{aligned} B_{eff}=\frac{t_B}{T} \end{aligned}$$

where $t_B$ is the total time when both hands were simultaneously doing something productive, in other words the tools were visible and the surgical activities had a definable target.

### Validation analysis

Although the action–target pairs are technically based on objective definitions, in some situations determining the correct target could leave room for interpretation. To assess the sensitivity of the results to annotation errors, we created new datasets by introducing artificial noise to the original annotations. The noise was added by switching the target labels with some probability ranging from 10% to 100%, with 10% point increments. The action labels were not changed because, with few exceptions, it is clear if the tool is moving, held still, transporting, grasping or not visible. Using the noise datasets, we conducted the analyses that were done with the original dataset to determine the highest percentage of noise (representing annotation errors or disagreements arising from different interpretations) that would invalidate the results. The validation analysis is similar to the one used by Forestier et al. in [5].

**Table 3:** Similarity of experts and novices at suture level, with more positive values indicating closer similarity to experts and negative values closer similarity to novices. The p column indicates whether expertise had a significant effect on similarity. $\sigma ^2$ is the variance of the random effect of participant

Overall				
Tool (hand)	Group	Similarity (95% C.I.)	p	$\sigma ^2$
MF (left)	Expert	0.228 (0.167, 0.290)	***	0.06
	Novice	$-0.145$ ($-0.207$, $-0.084$)		
NH (right)	Expert	0.206 (0.157, 0.255)	***	0.01<
	Novice	$-0.101$ ($-0.150$, $-0.052$)		

$$^{*}$$p<0.1, $$^{**}$$p<0.05, $$^{***}$$p<0.01

### Statistical analysis

We used linear mixed effects models to model the effect of expertise on efficiency and similarity. Both of these are measured on a bounded interval, which could pose problems with heteroscedasticity. However, at suture level the observed values were far enough from the bounds that the effect of heteroscedasticity was small. This was confirmed with diagnostics plots and Levene’s tests for each model. At segment level, there was more variation in residuals between segments and skill; therefore, the segment-level models used segment- and skill-dependent variances for the random effects.

At suture level, the models were fitted with expertise as a predictor for both efficiency and similarity. Hypothesis testing was done using t tests with Satterthwaite’s method.

**Fig. 4:**
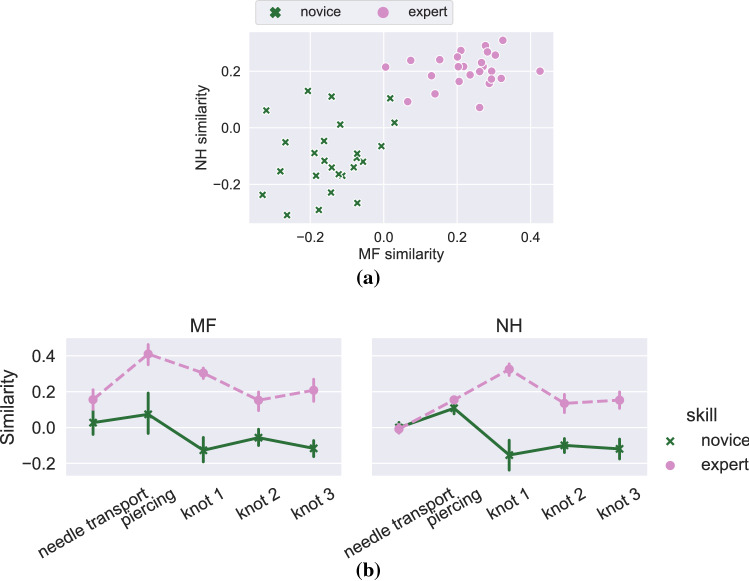
Overall similarity at suture level (**a**) and segment level (**b**), for the left hand tool (microforceps, MF) and the right hand tool (needleholder, NH). More negative values indicate closer similarity to novices and positive values closer similarity to experts

**Fig. 5:**
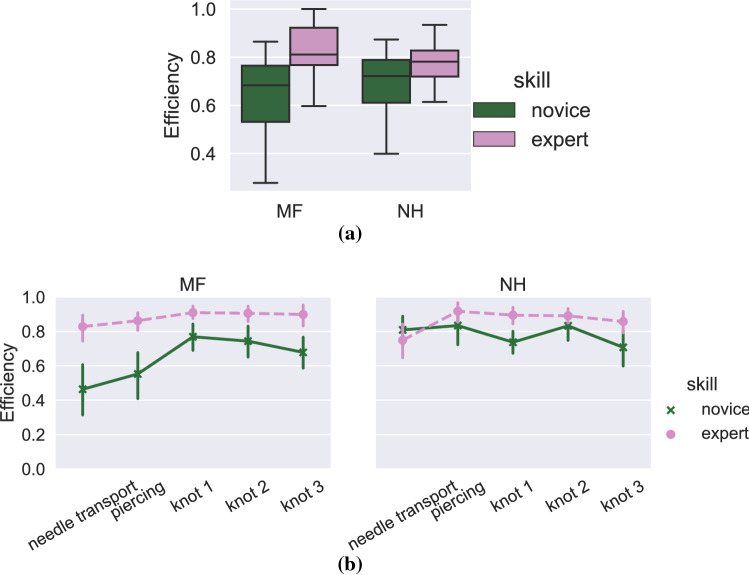
Left hand tool (microforceps, MF) and right hand tool (needleholder, NH) suturing efficiency at suture level (**a**) and at segment level (**b**)

At segment level, we are not interested in predicting the values for each segment, but in establishing whether segment and expertise have an interaction effect on similarity or efficiency. Thus at segment level, the hypothesis testing was done with type III ANOVA.

For both suture- and segment-level models, we included a random intercept for each participant to account for repeated measures. We compared visually the results of the two left-handed participants within their respective expertise groups and saw no indication that the handedness impacted their results.

Annotations, pre-processing of the data and the statistical analyses were done in Python using the Pandas data analysis library [20] and R [24] using the lme4 package [1] and the lmerTest package for hypothesis testing [16].

## Results

Of the 11 participants, one expert participant had to be discarded due to equipment failure. From the remaining 10 participants, we annotated the first 5 sutures where all the segments were completed successfully. However, for two novice participants we had to compromise by including three sutures without the third knot, because the sutures were otherwise successful, and other sutures from these participants had other issues. A fully completed suture had 5 segments (Table 1). Between piercing and the first knot (segments 2–3), as well as after the third knot (segment 5), there were parts where majority of the movement was outside the camera’s field of view, and these parts were not included in the analysis. In total, the final dataset contained 50 sutures and 247 segments for both hands, or a total of 494 annotated segments.

### Similarity

At suture-level, the experts were significantly more similar to other experts and novices were closer to other novices in both the microforceps and needle holder activities (Table 3).

At segment level, the interaction effect of segment and skill on similarity was significant ($F = 7.558$, $p<0.001$) as well as the main effects of skill ($F = 36.336$, $p<0.001$) and segment ($F = 14.059$, $p<0.001$) for the microforceps activities. For needleholder the interaction effect (F = 36.936, p<0.001) and the main effects of skill ($F = 62.205$, $p<0.001$) and segment ($F = 12.694$, $p<0.001$) were also significant. Figure 4a, 4b show the mean expert and novice overall similarities for sutures and segments, respectively.

### Suturing efficiency

Suturing efficiency was measured as the percentage of productive movements (tools were visible and the activity had a target). At suture level, the results indicated that novices have a lower efficiency with the microforceps ($\beta$ = -0.200, 95% C.I. [$-0.347$, $-0.052$], variance of the random effect of participant $\sigma ^2 = 0.111^2$) but not with the needleholder ($\beta$ = -0.095, 95% C.I. [$-0.178$, $-0.013$], variance of the random effect of participant $\sigma ^2 = 0.052^2$). At segment level, the interaction effect of skill and segment was significant for the microforceps ($F = 3.196$, $p = 0.014$), as well as the main effects of skill ($F = 5.580$, $p = 0.046$) and segment ($F = 9.465$, $p<0.01$). For needleholder, the interaction effect was significant ($F = 3.073$, $p=0.017$) as well as the main effect of segment ($F = 2.659$, $p = 0.033$), but the effect of skill was not significant, ($F = 4.198$, $p = 0.075$). On average, the experts had 29.3 % higher efficiency in their microforceps movements, and 12.6 % higher efficiency in their needleholder movements, or 21.0 % higher efficiency on average. Figure 5a, b show the overall efficiency for novices and experts at suture and segment levels.

### Bimanual efficiency

Bimanual efficiency was defined as the percentage of the total suturing duration when the participants simultaneously used both hands with a target. For sutures, the novice bimanual efficiency was lower ($\beta = -0.255$, 95% C.I. [$-0.438$, $-0.072$], variance of random participant effect $\sigma ^2 = 0.142^2$).

**Fig. 6:**
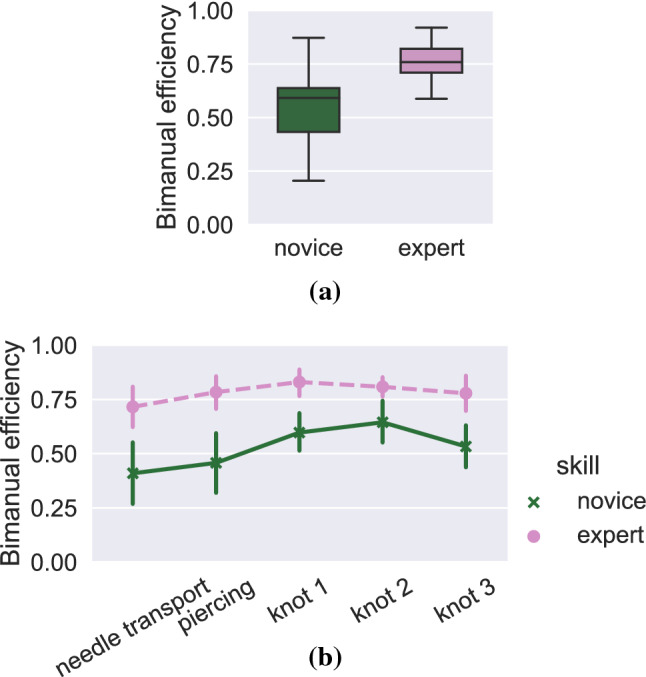
Comparison of bimanual efficiency for sutures (**a**) and segments of suturing (**b**)

At segment level, type III ANOVA indicated a significant effect of skill ($F = 7.244$, $p = 0.027$) and segment ($F = 4.961$, $p< 0.01$), but the interaction effect of expertise and segment was not significant ($F = 1.130$, $p = 0.343$). On average, the experts had 48.2 % higher bimanual efficiency. Figure 6a, b show the bimanual efficiency for novices and experts at suture and segment levels.

### Validation results

For the similarity analysis, the validation analysis showed that even with the noise dataset where 100% of the target labels were changed, the differences between novices and experts remained statistically significant at suture level for microforceps (expert similarity = 0.047, novice similarity = $-0.043$, difference statistically significant with $t=-2.985$, $p = 0.018$) and the needleholder (expert similarity = 0.011, novice similarity = $-0.135$, $t = -7.931$, $p < 0.001$). At segment level, the results for microforceps were significant for skill ($F = 7.358$, $p = 0.027$) and segment ($F = 28.521$, $p < 0.001$), but not their interaction ($F = 1.792$, $p = 0.131$). For needleholder, the results were significant for skill ($F = 29.813$, $p < 0.001$), segment ($F = 2.648$, $p = 0.034$) and their interaction ($F = 13.770$, $p < 0.001$).

The results imply that the differences were based more on the actions, and that novices and experts who participated in this study could be distinguished even without considering the targets. To test this, we calculated the similarity using only the four actions, tool not visible and idle time. The results at suture level were significant for microforceps (expert similarity = 0.124, novice similarity = 0.011, $t = -2.840$, $p = 0.022$) and needleholder (expert similarity = 0.215, novice similarity = $-0.172$, $t = -12.439$, $p<0.01$). Because even determining the idle time can require some interpretation of the ongoing task, we also calculated the similarities using only the four actions, which can be most objectively seen from the videos. The results at suture level remained significant for the needleholder (expert similarity = 0.196, novice similarity = $-0.169$, $t = -11.550$, $p<0.01$), but not for the microforceps (expert similarity = 0.052, novice similarity = 0.053, $t = 0.054 p = 0.958$).

The difference in microforceps suturing efficiency remained statistically significant in the dataset where 60% of the target labels were changed (novice efficiency $-0.1648$, $t = -2.567$, $p = 0.0333$. At segment level, the efficiency difference remained statistically significant in the dataset where 30% of the target labels were changed for the main effect of skill ($F = 6.265$, $p = 0.0367$), segment ($F = 5.596$, $p<0.001$) and their interaction ($F = 3.165$, $p = 0.015$). Difference in bimanual efficiency remained statistically significant with the dataset where 30% of the labels were changed (novice efficiency $-0.159$, $t = -2.686$, $p = 0.028$). At the same noise limit, the segment-level differences were significant for skill ($F = 5.596$, $p = 0.046$) and segment ($F = 3.141$, $p = 0.015$), but not for their interaction ($F = 2.061$, $p = 0.087$).

## Discussion

In this paper, we proposed and defined a new movement-level surgical process model and demonstrated how the model reveals important differences in novice and expert surgeons’ uni- and bi-manual efficiency during a microsurgical procedure. We were able to show that there is a clear difference in how efficiently the novices and experts used their instruments, and that the participants’ level of expertise can be differentiated by their movement patterns even when the surgical process model is defined with minimal number of movement types.

By measuring the cosine similarity between surgical process model descriptions of tool usage, we were able to show significant differences between novices and experts. Our results indicate that the movements with the microforceps (left hand tool) were consistently different between the two groups, and that the needleholder movements diverged more during knotting (Fig. 4b). Using a similar surgical process modeling approach than the one described here, Uemura et al. [26] found that the differences between novices and experts were larger for the left hand. A notable result is that it is the similarity to experts that actually separates novice and expert surgeons. The likely reason is that the novices’ movements are less consistent, i.e. there is no ”typical novice,” and therefore, no participant strongly resembles the novice, regardless of skill.

Our suturing efficiency results show that 32.0 % of the novices’ microforceps movements were nonproductive (Fig. 5a). Uemura et al. found that the novices spent more time without the hands engaged in productive activities (which they termed *dwell time*) during a laparoscopic training task. [26] Their results indicated that novices spent on average as much as 18.65 % of the task duration on dwell time. Our definition included the time when the tools were not visible, with the assumption that the participants would not be doing anything productive without seeing their tools. However, in some parts of the task the tool had to be moved out of view, for example, when the thread is being extracted after piercing.

The results on bimanual efficiency align with prior studies that focused on other surgical settings and used different approaches to movement analysis. Hofstadt et al. defined *bimanual dexterity* as the correlation between non-dominant and dominant hand instrument velocities and found that experts had significantly higher correlations than novices. Likewise, they found that experts required fewer sub-movements and were better than novices at using their non-dominant hand, indicating better efficiency similar to our results [12]. Other studies have also reported that novices tend to neglect their non-dominant hand in laparoscopic tasks [14, 15, 18]. Zulbaran-Rojas et al. reported that the novices’ non-dominant hand had not only less activity than the dominant hand (measured by velocity and traveled distance), but also more wasted movements, and suggested that the ability to use both hands equally is a sign of expertise [30]. Similar findings have been reported by Uemura et al., who defined a novel surgical skill score metric based partially on bimanual movements and found that experts were better at coordinating their movements bimanually [27]. Though the definition of bimanual dexterity in these studies differs from ours, their findings agree with our results.

A segment-level comparison of efficiency and similarity showed that the differences between novices and experts varied depending on the phase of the task (Figs. 4b, 5b, 6b). Earlier research has shown that surgical trainees find some parts of surgical procedures more difficult than others [4], confirmed also by pupillary-response studies [2]. In [29], the authors compared novice, intermediate and expert participants’ efficiency at task and segment levels, and found that that the novice and intermediate participants were less efficient in all segments, echoing our results (Figures 5b and 6b). Interestingly, our results (see Fig. 5b) show that the experts’ efficiency in the first segment is actually slightly lower than the novices’, though the difference is not significant. This can be explained by the fact that the experts took more care to adjust the needle’s position properly before insertion, and even though these movements are important for ensuring a high quality suture, the extra movements during adjustment may contain movements that are counted as nonproductive.

Previous studies have defined surgical process models using several different actions, targets/structures and instruments. For example, Uemura et al. [26] used nine actions, six instruments and four different structures. In the recent MISAW challenge [13], where the goal was to recognize surgical workflow at different granularity levels, the surgical activities were defined using ten actions (”Verbs”) and nine targets. Our results indicate that a model defined even with extremely limited vocabulary can still differentiate novices from experts in a simulation training task. The MISAW challenge results showed that, of the different granularity levels, activities were hardest to recognize. A limited vocabulary of activies such as the one used in this work might be easier to recognize while still being useful for extracting basic information about the participant’s surgical performance.

One limitation of this study is the fact there are subtle mistakes that the surgical process model cannot reveal. The small number of activities used to define the model may lead to same similarity values for procedures even when the precision of the movements—for example during piercing—is different. This limitation is to some extent inherent to any surgical process model; to detect the subtler mistakes would require supplementing the surgical process analysis with other sources of data. Here, we also compared novices whose movements may be easily distinguishable from experts. Whether this method would be able to detect differences between experts and intermediate participants has to be investigated in future studies.

Another possible limitation related to the choice of granularity is that when the elementary activities are defined at basic movement level, the transition from one activity to another can become less clear, and at the same time the duration of the individual activities becomes shorter—which means that errors in the annotations could affect the results. To evaluate the results’ sensitivity to annotation errors, we conducted several validation tests with artificial noise datasets. The validation results showed that even with serious disagreements between annotations, the novices and experts were still distinguishable. In fact, some differences remained significant even with a bare-bones model consisting only of the four actions.

The surgical process model defined here combined two different approaches. First, it consists of the top-down description of surgical segments, which have a fixed order and whose definition requires higher-level information of the entire surgical process. Second, the model includes the bottom-up description of elementary surgical activities, determined using low-level information from short clips of the surgical process. The elementary surgical actions comprised of a few basic actions and targets, yet allowed the computational assessment of the surgeon’s bimanual dexterity and movement patterns not only at suture level, but also in different segments of the suture. Constructing the model in this manner means that the numerical results can be more easily translated into qualitative feedback. For example, a segment level comparison of similarity between surgical trainee’s suture and expert’s suture could show that they differed mostly in the beginning of the suture, and comparing the activities in this segment could show that the difference arose mainly because the novice failed to use the left hand tool efficiently. Although in our case the elementary actions were analyzed manually by a human observer, the simplicity of the actions facilitates their automated detection.

Future work requires the development of an automated method for detecting the surgical activities. In microsurgery, one way of accomplishing this would be by applying computer vision methods to the videos recorded from modern surgical microscopes. Automatic detection of the surgical activities would allow the comparison of surgical trainees’ performance to that of typical expert surgeons. Comparing the similarities at segment level will help the trainees to pinpoint troublesome parts of the procedure, in other words they could be provided with automatic feedback that does not require expert intervention. During clinical surgery, detection of overt differences of surgical actions when compared to experts performing a similar case—or even to the surgeon’s own previous cases—could provide a safety measure by detecting when the procedure is deviating from safe conduct.
